# High sensitivity detection of extracellular vesicles immune-captured from urine by conventional flow cytometry

**DOI:** 10.1038/s41598-019-38516-8

**Published:** 2019-02-14

**Authors:** Carmen Campos-Silva, Henar Suárez, Ricardo Jara-Acevedo, Estefanía Linares-Espinós, Luis Martinez-Piñeiro, María Yáñez-Mó, Mar Valés-Gómez

**Affiliations:** 1 Department of Immunology and Oncology, National Centre for Biotechnology, CNB-CSIC, Madrid, Spain; 2 Department of Molecular Biology, UAM, Centro de Biología Molecular Severo Ochoa (CBM-SO), Instituto de Investigación Sanitaria Princesa (IIS-IP), Madrid, Spain; 3 Immunostep, S.L., Salamanca, Spain; 4 Servicio de Urología and Instituto Sanitario (Idipaz), Hospital Universitario La Paz, Madrid, Spain

## Abstract

Extracellular vesicles (EVs) provide an invaluable tool to analyse physiological processes because they transport, in biological fluids, biomolecules secreted from diverse tissues of an individual. EV biomarker detection requires highly sensitive techniques able to identify individual molecules. However, the lack of widespread, affordable methodologies for high-throughput EV analyses means that studies on biomarkers have not been done in large patient cohorts. To develop tools for EV analysis in biological samples, we evaluated here the critical parameters to optimise an assay based on immunocapture of EVs followed by flow cytometry. We describe a straightforward method for EV detection using general EV markers like the tetraspanins CD9, CD63 and CD81, that allowed highly sensitive detection of urinary EVs without prior enrichment. In proof-of-concept experiments, an epithelial marker enriched in carcinoma cells, EpCAM, was identified in EVs from cell lines and directly in urine samples. However, whereas EVs isolated from 5–10 ml of urine were required for western blot detection of EpCAM, only 500 μl of urine were sufficient to visualise EpCAM expression by flow cytometry. This method has the potential to allow any laboratory with access to conventional flow cytometry to identify surface markers on EVs, even non-abundant proteins, using minimally processed biological samples.

## Introduction

Most cell types release extracellular vesicles (EVs) during physiological processes. There exist different types of EVs, among which the term exosomes refers to nanovesicles (30–200 nm) released after fusion with the plasma membrane of intraluminal vesicles enclosed in endocytic compartments known as multivesicular bodies (MVB)^1,2^. Other types of EVs include microvesicles, which are usually larger than exosomes (200 nm-1 µm) and do not originate from the endocytic pathway, instead they bud from the plasma membrane^3^. There are several databases including information on the content of EVs: Exocarta^4^, EVPedia^5^, Vesiclepedia^6^, however, recent data have revealed that there is a great degree of heterogeneity among EVs and they exhibit different markers depending on the mechanism of release and the cellular origin^7^. Nanovesicles can be found in the extracellular milieu, like tissue culture supernatant, but also in biological fluids, like plasma and urine, and they carry many types of biomolecules, including proteins, lipids, mRNA, miRNA and DNA^8^. Therefore, EVs can mediate intercellular communication and macromolecules transfer and they also provide information about patho-physiological processes happening in an individual. Because EVs can be found in blood and urine, they have attracted much interest as potential biomarker targets and they are included in the recently coined term, liquid biopsy. This expression was initially used to refer to the analysis of the tumour burden by examining circulating tumour cells (CTCs) or DNA (ctDNA)^9^. Nowadays much research effort is being invested to understand the biological roles of circulating EVs, to identify their origin (distinguishing those from healthy cells from those associated with pathology) and to unveil their use as biomarkers. Progress in these research areas depends on the ability to systematically characterize EVs using standard, quantitative methods that allow comparison of results obtained in different laboratories and hospitals. The ideal new diagnostic tool should use small sample volumes of blood or any other biological fluid for monitoring of the disease, allowing the generation of results from many samples in a laboratory user- friendly setting. Several methods are currently used for EV enrichment before further characterization; for example, serial ultracentrifugation steps^10^, precipitation^11^, density gradient separation or size exclusion chromatography^12–14^. Each of these techniques has advantages and disadvantages in terms of purity or enrichment of EVs and the decision to utilize one or the other depends on the downstream use envisaged for the sample recovered and the importance of the impurities or co-isolated material found in each case. Size and concentration are currently measured by physical methods, such as nanoparticle tracking analysis (NTA) or conventional protein concentration tests, while their protein or nucleic acid content can be analysed by conventional laboratory methods like Western Blot and PCR. However, most of these methods for enrichment and characterization are expensive and time consuming and essentially make impossible the screening of a large number of samples.

An important step in EV characterization relies on determining the molecular composition of vesicles and identifying markers of disease. Choosing universal exosome markers is challenging because of cell-to-cell variability and differential expression in different types of EVs. Moreover, there is little information about how biological processes, such as tumor transformation, affect the relative amount of protein markers recruited into EVs. However, recent data comparing the composition of EVs isolated after different centrifugation speeds (2000 × g, 10,000 × g and 100,000 × g pellets) make it clear that CD63, CD9, CD81 or combinations of these molecules are enriched in EVs derived from different cell lines, although these preparations may also contain non-EV material co-purified with EVs^7^. Depending on the cell origin, the 100,000 × g pellet can contain small (30–50 nm) or larger (50–200 nm) vesicles^2^. Thus, immunocapture, using tetraspanins CD63, CD9 and CD81, or other molecules generally found in EVs, such as TSG101, Alix, etc^15^, can provide a tool to selectively enrich EVs from a complex preparation. Although there are some studies reporting successful immunocapture of EVs, the assay conditions need to be individually optimised depending on the readout technique that will be used afterwards and implementation of the methodology varies significantly among different laboratories. For example, we and others have reported EV detection using enzyme-linked immunosorbent assays (ELISA)^16–18^ or lateral flow immunoassays (LFIA)^17,18^ demonstrating that each one of these techniques has different critical steps affecting sensitivity. Novel techniques using immunocapture-based microfluidics^19^ or time-resolved fluorescence immunoassay (TR-FIA)^20^ can also provide a very good research tool for EV screenings, however the use of specialized equipment and appropriately trained operators is required. Immune-capture followed by flow cytometry detection, in general requires immobilization of the nano-sized EVs on microbeads; although a range of differently sized vesicles are released by cells, average exosomes have a diameter of around 100 nm and the laser beam of the flow cytometer does not resolve light scattered by particles smaller than 300 nm (these particles fall together with cell debris, protein or antibody aggregates in FSC plots), unless a special flow cytometer with a modified laser is used^21–24^. For conventional flow cytometry, microbeads with different composition (latex, polystyrene, etc), different sizes (4–9 microns in diameter) and different functionalization (antibodies, streptavidin, aldehyde sulphate) have been already employed^25–28^. All these studies demonstrate that, once the size limitation is overcome by coupling to microspheres, EVs can be visualised by flow cytometry allowing specific analysis of proteins on their surface. However, in all these settings either large amounts of starting material or previously enriched EVs were required. With the aim of establishing a standard reproducible methodology for detection of EVs by flow cytometry and to improve available methods for EV characterization, here, we defined the critical parameters necessary to increase EV detection capacity by flow cytometry, after immune-capture on magnetic fluorescent beads coated with tetraspanin-specific antibodies. We have defined the conditions to achieve very high sensitivity detection of EVs, so that biological samples can be analysed with minimal sample processing. In fact, we could detect CD9-containing vesicles directly in as little as 500 µl of urine from healthy donors after capture on anti-CD63-coated beads. Furthermore, extracellular vesicles captured on anti-EpCAM coated beads were detected directly in urine.

## Results

### Characterization of EVs from the prostate cell line PC3

**Figure 1:**
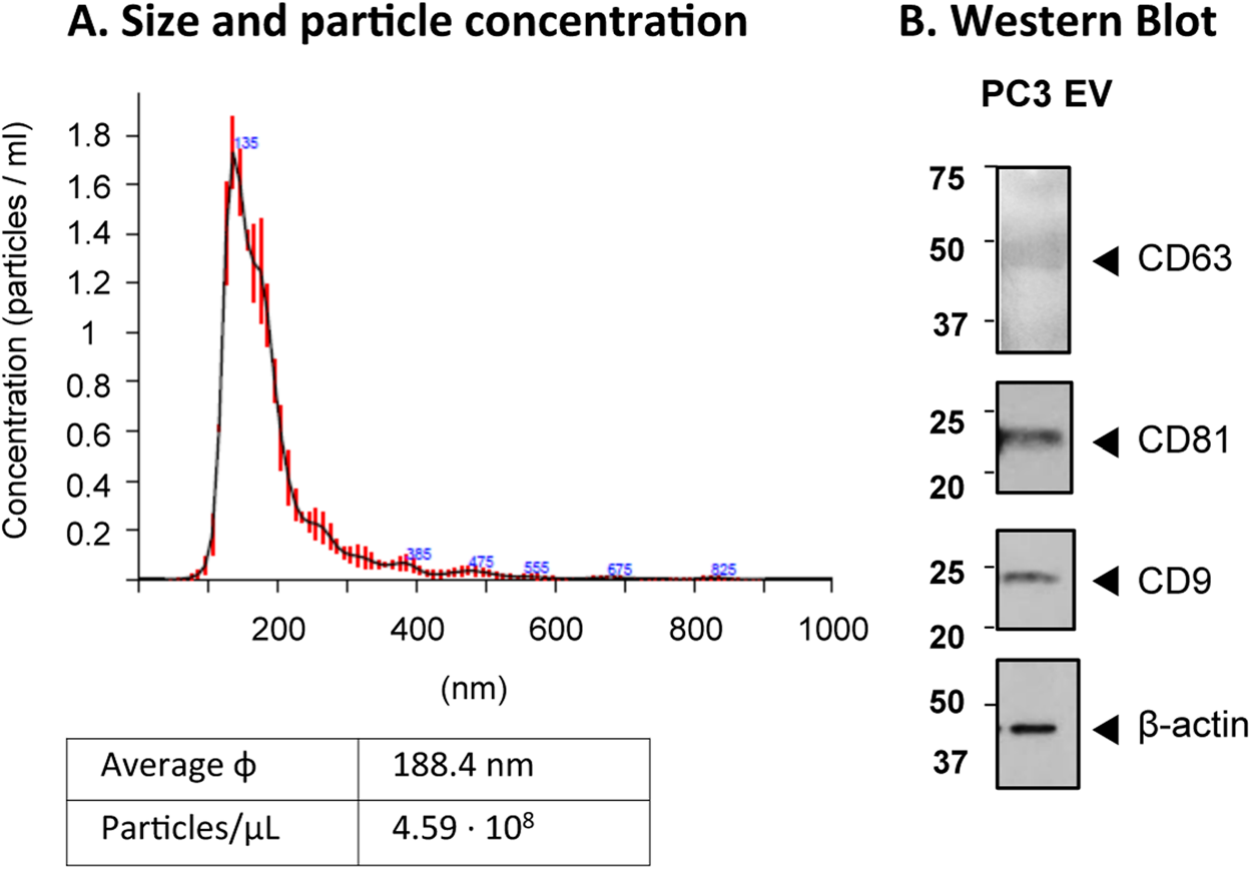
Characterization of PC3-derived EVs. (**A**) Nanoparticle tracking analysis (NTA). Average size and concentration were obtained in a Nanosight equipment capturing 3 videos of 60 s per measurement, with a focus −15 to +15 and camera level 12. ɸ: diameter. (**B**) Western Blot. EVs were loaded on SDS-PAGE and immunoblotted for β-actin (Sigma) and antibodies against tetraspanins [anti-CD9 (MEM62), -CD63 (MEM259) and -CD81 (MEM-38)]. Three gels were loaded: one gel, under non-reducing conditions with 2.2·10^9^ particles, for CD9 and CD81 detection, exposed for 2 min; a second non-reducing gel with 6.8 · 10^9^ particles, for CD63 detection, exposed for 1 h; and the third gel under reducing conditions, for actin detection, exposed for 40 s. The experiment shown is representative of 4.

To improve the methodology for optimal EV immune capture on magnetic microbeads, followed by flow cytometry detection, we focused on the study of vesicles from a broadly used cell line from prostate cancer, PC3. Initial experiments for optimization of the procedure were carried out using PC3-derived EVs, either commercially available or purified in our laboratory by sequential ultracentrifugation and preserved by lyophilization. Firstly, EVs were characterised by NTA and Western Blot (Fig. 1A,B; Supplementary Fig. 1). Reconstituted EVs showed by NTA a mean size of 188.4 +/− 1.9 nm (Standard error; SD: 86 nm), a concentration of 4.59∙10^8^ +/− 1.49∙10^7^ particles/ml and contained the common markers of EVs; the tetraspanins CD63, CD81 and CD9. Moreover, the EV fraction from PC3 was enriched in CD9, compared to the cell lysates and did not contain calreticulin, an ER marker, present in cell lysates (Supplementary Fig. 1). In addition, PC3-derived EVs were positive for EpCAM, an epithelial cell marker.

### Theoretical considerations for immune capture of EVs on microbeads

**Figure 2:**
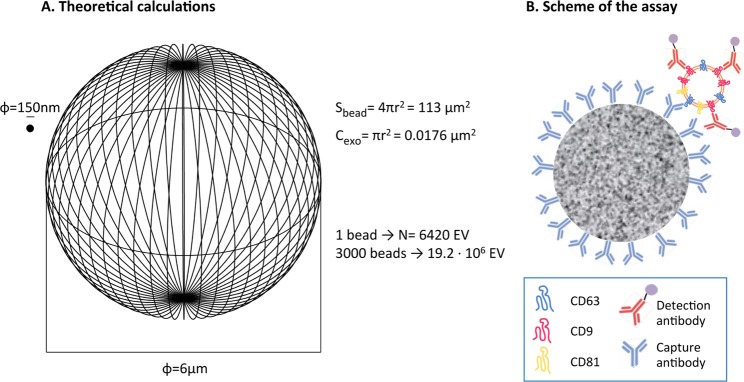
(**A**) Theoretical calculation of the number of EVs immobilised per bead. The graph represents at real scale a 6 μm-diameter bead and an EV of 150 nm of diameter (ɸ). The external surface of a bead (S_bead_) could capture a maximum of N times the circle area (C_exo_) of 150 nm EVs. N is calculated by dividing S_bead_/C_exo_. (**B**) Schematic representation, not to scale, of the bead binding assay. 6 μm-diameter fluorescent magnetic beads were coated with capture antibody and used for immune-capture of the EVs. A second antibody directed against the same or a different molecule, either biotinylated or directly conjugated with PE, was used for detection. In the case of biotinylated antibodies, PE-conjugated streptavidin was subsequently used. Samples were analysed by conventional flow cytometry.

To establish the conditions for a high sensitivity method of EV immune-capture, we first considered which parameters are critical to get the best signal-to-noise ratio performance in a flow cytometer. Because there is a linear relationship between the intensity of fluorescence detected in a flow cytometer and the amount of a given fluorochrome per cell (in our case, per bead)^29^, the fluorescence signal will be maximum if the number of EVs captured per bead tends towards saturation. Thus, for optimal signal detection two parameters need to be carefully established: (1) the total number of beads acquired per experiment, and (2) the number of EVs that contain the epitope used for capture. Moreover, since our goal is to analyse biological samples, the optimization has to take into account that EVs could be diluted in a large volume, for example, in urine and, in this case, the dilution factor could be important. In other biological samples, such as plasma or serum, EVs derived from unhealthy cells or tumours could be mixed with large amounts of EVs released by normal tissues (as well as all the other usual components of human serum). Thus, non-abundant markers for tumour or stress would be needed to capture the EVs of interest. So, given that the relevant EVs can be in limiting amounts in biological fluids, the assay should be optimised using the minimal number of beads that allows reproducible statistical analysis of the events acquired by the flow cytometer. For a better understanding of the system, we estimated the maximum binding capacity per bead, assuming that the whole surface of the bead would be covered with antibody and that a monolayer of EVs would be coupled to the bead. The maximum number of EVs captured per bead was calculated superimposing on the surface of a sphere (S_bead_) the number (N) of EV circle surfaces (C_exo_, taking as average diameter, 150 nm) which could fit in that area; N was calculated as the ratio N = S_bead_/C_exo_ (Fig. 2). This average diameter was taken because, in our previous experiments^18^, the average size of exosomes purified by ultracentrifugation was nearly 200 nm by NTA, but EM pictures revealed particles near or below 100 nm, and this was also true for the EVs used here. According to this calculation, a 6 μm bead would bind a maximum of 6420 EVs. If the cytometer acquired 3000–6000 beads per test, a sample with 1.93–3.85 × 10^7^ EVs would saturate the beads. These calculations assume that all the EVs in the mix would be able to get in contact with the beads, however, beads and EVs in solution have different physical behaviour due to their difference in size and density, so that the beads can precipitate to the bottom of the tube while EVs would remain in suspension longer time. On the other hand, the number of antibody molecules reported to bind T lymphocytes under saturating conditions, for bright epitopes such as CD4, has been reported to be around 2–4 ∙ 10^4^ ^30,31^. Thus, the fluorescence intensity should be detectable even in the case that only half of the binding surface of the bead was covered by EVs, that is, around 3200 EVs, if each EV was recognised by 6–12 molecules of detection antibody.

### Specificity of immunocapture using anti-tetraspanin antibodies

**Figure 3:**
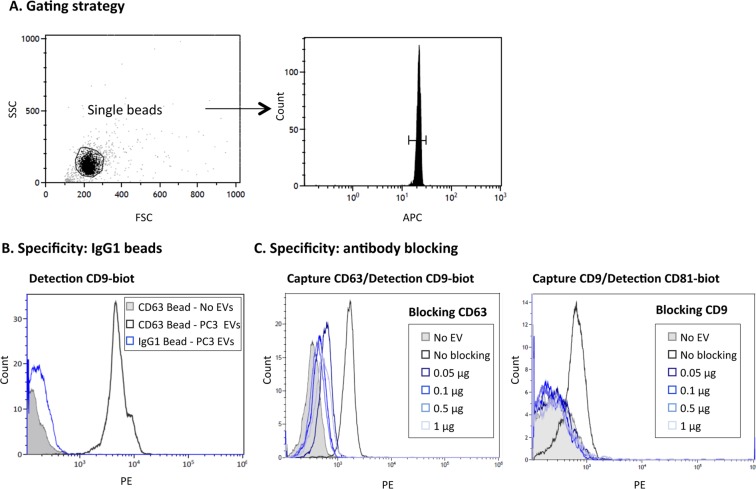
Specificity of EV immunocapture on antibody-coated microbeads. (**A**) Gating strategy. EVs immobilised on 6 μm APC-beads were stained using biotinylated antibody followed by PE-conjugated streptavidin and analysed by flow cytometry. A gate containing only single beads was created in the Forward Scatter (FSC)/Side Scatter (SSC) plot. A second gate, within single beads, confirmed the APC fluorescence of microbeads. 1500–2000 events from this combined gating were acquired and analysed for PE labelling. (**B**) Negative control, IgG1. 10^9^ particles of PC3-derived EVs were captured onto either anti-CD63 (Clone TEA3/18) or IgG1-coated beads followed by detection with biotinylated antibody directed against CD9. A sample with no EVs is also shown for comparison. (**C**) Antibody blocking. 10^9^ particles of PC3-derived EVs were pre-incubated with increasing amounts of the indicated soluble blocking antibody [anti-CD63 (Clone TEA3/18), anti-CD9 (Clone VJ1/20)] before being incubated for capture on CD63- (left) or CD9- (right) coated beads. Experiments are representative of 3 independent repetitions.

The assay was designed using 6 μm antibody-coated fluorescent magnetic microbeads as depicted in Fig. 2B. Taking into account previous calculations, the starting point was established as 2 µg of EVs from PC3 (10^9^ EVs), which should provide a good excess for capture with 3000–6000 beads, even in the case that not all the EVs in the sample expressed the epitope used for immunocapture. The gating strategy excluded bead aggregates and debris by FSC/SSC and selected the acquisition number in the region of APC-positive microbeads (Fig. 3A). To confirm the specificity of the recognition, microbeads covered with irrelevant antibody (murine IgG1 control protein) were used to compare flow cytometry signals (Fig. 3B). Capture conditions were optimised trying different volumes, temperatures and times of incubation followed by detection with PE-conjugated anti CD81 antibody (Supplementary Fig. 2). A stronger signal was detected when EVs were incubated without agitation with 6000 antibody-coated beads for 18 h at RT. Antibody blocking experiments were also performed including a sample pre-incubation step with the same antibody used for immunocapture (either purified anti-CD63 or anti-CD9 antibodies) (Fig. 3C). 50 ng of antibody were sufficient to completely block binding of the EVs to the microspheres.

### EV detection using CD63 antibody for capture. Limit of detection

**Figure 4:**
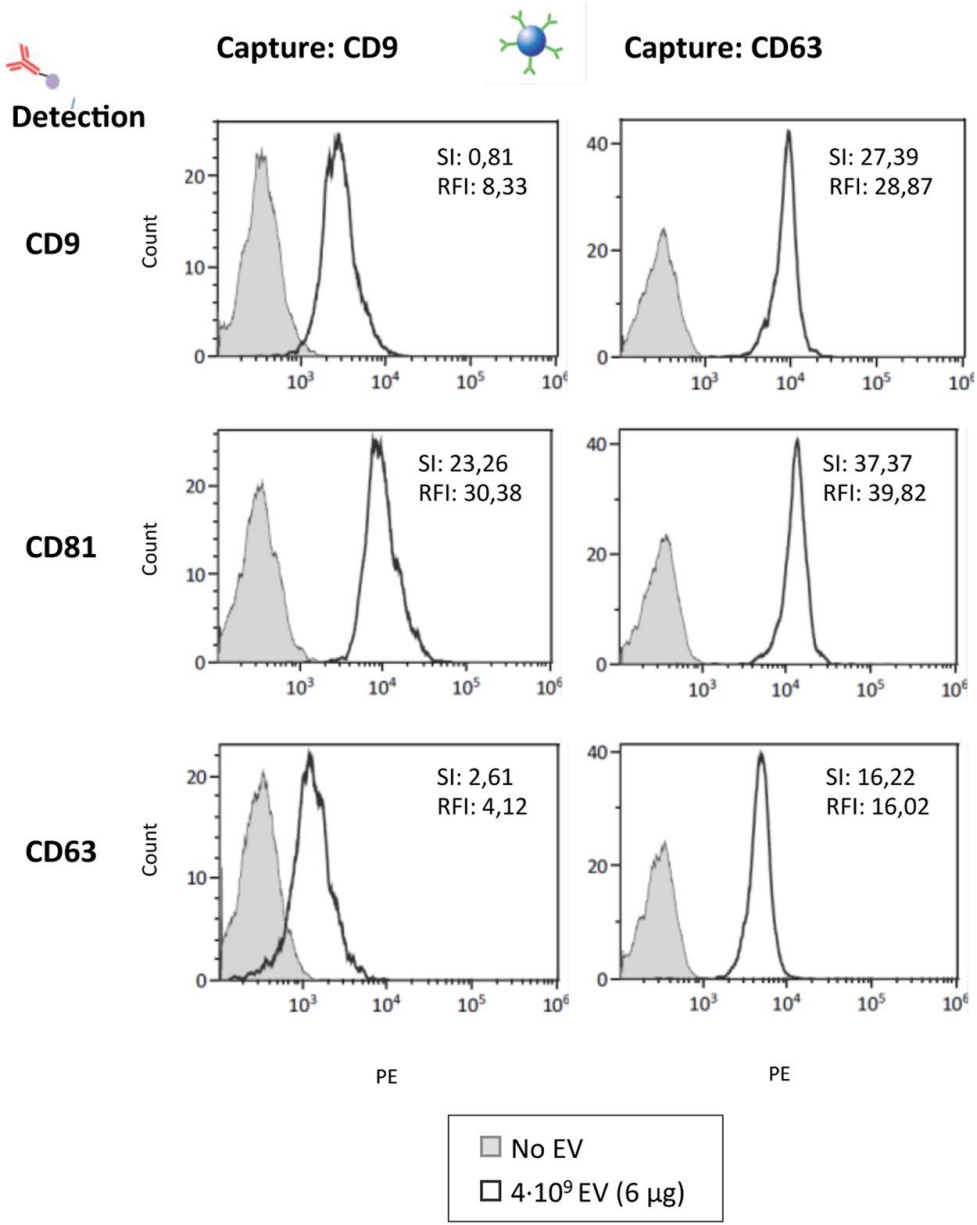
Optimization of tetraspanin antibody combination. 4∙10^9^ particles of PC3-derived EVs were captured onto either anti-CD9 (Clone VJ1/20) or anti-CD63 (Clone TEA3/18) coated beads followed by detection with PE-conjugated antibodies directed against CD9, CD81 or CD63. The sensitivity of each antibody combination is compared using the Stain Index (SI) $SI=\frac{MFI\,positive\mbox{--}MFI\,background}{2\sigma \,background}$, where σ is the standard deviation and MFI Mean Fluorescence Intensity; and the Relative fluorescence Index (RFI), $RFI=\frac{MFI\,positive}{MFI\,background}$, indicated on the upper right corner of each panel. A representative experiment out of 3 is shown.

CD63, CD9 and CD81 are general markers of EVs, however, these tetraspanins can be present in different amounts on EV subpopulations. In addition, different antibodies against different tetraspanins provide another source of variation depending on affinity of the interactions. Thus, we determined the optimal combination of tetraspanin capture and detection antibodies for flow cytometry visualization of PC3 EVs. Anti-CD9 antibody or anti-CD63 antibody coated magnetic microspheres were incubated for 18 h with PC3 EVs (or without EVs for negative control) and thereafter stained with either anti-CD63, anti-CD9 or anti-CD81 antibodies for detection. The number of EVs (4 × 10^9^ particles) used in each one of the assays was kept constant. The best efficiency (positive vs negative signal and highest absolute number) was observed using anti-CD63 antibody coated magnetic microspheres for capture (Fig. 4). To compare the relative staining obtained with the different antibody combinations, the fluorescence intensity was evaluated taking into account the peak distance between the positive sample and the background as well as the spread of the negative. Thus, the Relative Stain Index (SI) was calculated according to the formula, $SI=\frac{MFI\,positive\mbox{--}MFI\,background}{2\sigma \,background}$. An alternative measurement of relative brightness is the signal-to-noise ratio, Relative Fluorescence Intensity (RFI), calculated as RFI $=\frac{MFI\,positive}{MFI\,background}$ ^32,33^. Results show that, although CD63 was in general a less abundant protein than CD9 or CD81 in PC3 EVs, as judged by the higher time of exposure required for CD63 in WB experiments, the CD63-specific mAb was suitable for capture yielding high SI and RFI when either CD9 or CD81 mAbs were used for detection. In contrast, CD63 was not as efficient when used as detection antibody. Using CD63 for capture, allowed to further amplify the signal using fluorescent streptavidin binding biotinylated antibodies against the abundant tetraspanins CD9 and CD81. (Supplementary Fig. 3A).

**Figure 5:**
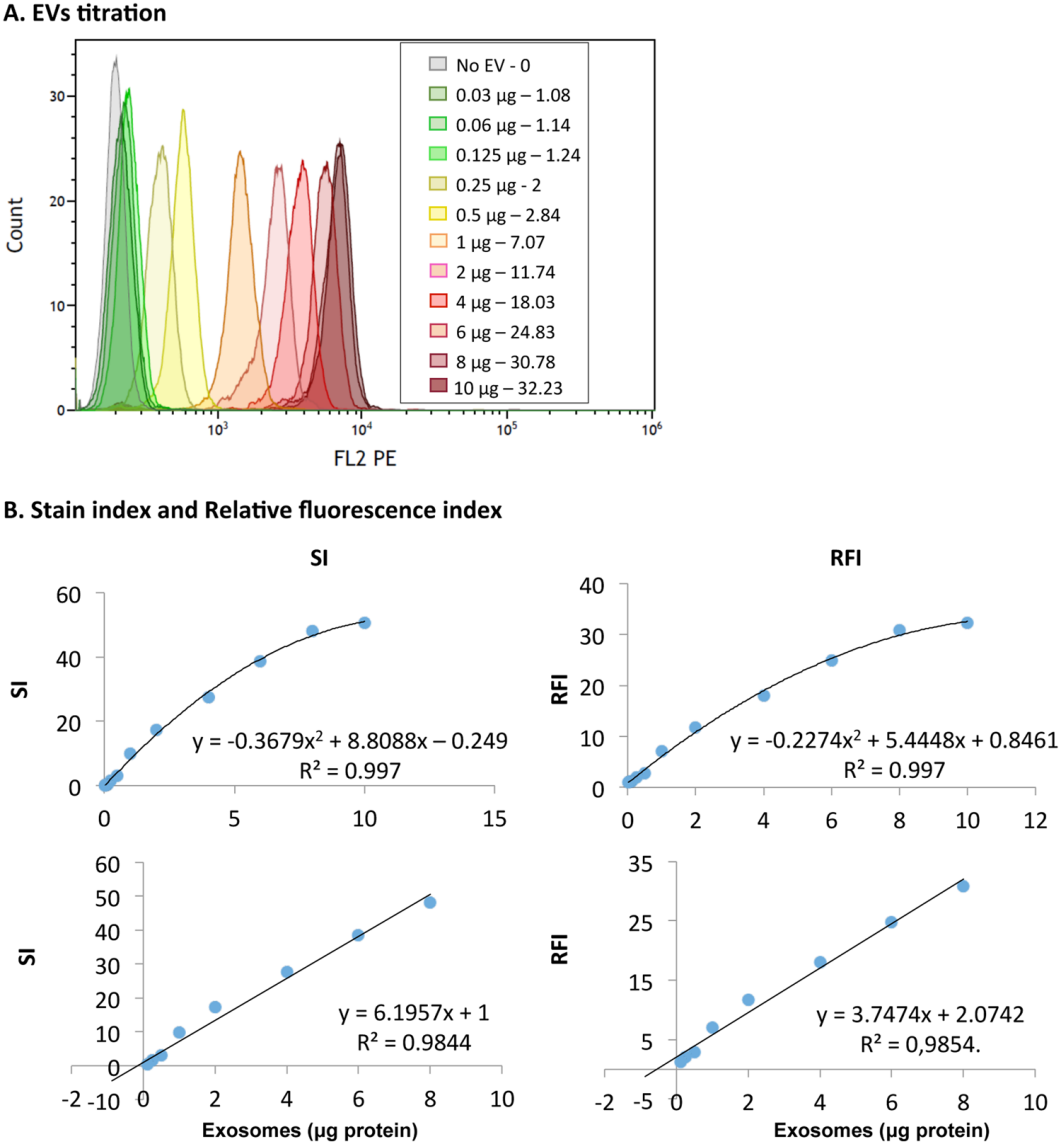
Dynamic range and limit of detection. EVs were captured on anti-CD63-coated beads followed by detection with biotinylated anti-CD9 antibody (Clone VJ1/20). (**A**) Flow cytometry analysis profiles. Increasing amounts (30 ng to 10 μg; 1.37∙10^7^–4.59∙10^9^ particles) of PC3-derived EVs were captured on 6 μm beads and detected by flow cytometry. The total volume of the assay was 100 μl. The graph represents the overlay of the curves obtained. The legend indicates the amount of EVs and the RFI for each curve. (**B**) Regression analysis. RFI (right) and SI values (left) were plotted as a scatter graph and fitted to a polynomic curve (upper panel). Linearity can be observed between 0.2–8 µg of PC3-derived EVs with a r^2^ > 0.98 (lower panels). The minimal amount of EVs detected (RFI > 1), corresponds to 30 ng yielding a RFI of 1.08. A representative experiment out of 3 is shown.

Next, to determine the dynamic range and limit of detection of the assay, EV titration experiments were performed using CD63 for capture and CD9 for detection (Fig. 5A). Different amounts of EVs were also tried on IgG1-coupled beads with minimal increase in fluorescence intensities (For example, Supplementary Fig. 3B). The sensitivity of the assay was very high, with a positive signal detected when as little as 30 ng (1.37∙10^7^ particles) of EVs were incubated with the microspheres (Fig. 5B), while for detection of CD63 by Western Blotting 6.8 · 10^9^ particles (14.85 µg) were required. Flow cytometry detection data followed a linear distribution between 30 ng and 8 μg (R² > 0.98, for both RFI and SI), corresponding to 1.37∙10^7^–3.67∙10^9^ particles. The minimum amount of EVs necessary for a reliable detection can be defined by calculating the SI allowing complete separation of the 2 peaks, that is, when SI > 1, meaning that the two peaks would be completely resolved and separated by 2 times the standard deviation of the normal distribution. We could define the minimal value for a reliable limit of EV detection by flow cytometry as 142 ng of EV (7∙10^7^ particles), by interpolating in the polynomic curve when SI = 1.

To confirm that EVs from different sources could be detected using flow cytometry, EVs from different cell lines were enriched by sequential centrifugation from tissue culture supernatant. The different cell lines displayed varying amounts of CD63 and CD9 as detected by WB, however, immune-capture followed by flow cytometry detection was possible for all of them with higher sensitivity than WB (Supplementary Fig. 4). EVs from MCF-7, RT-112, SK-Mel-147, Ma-Mel-55, SK-Mel-28 cell lines could be detected (also from HEK293 and SUM149 not shown). SI and RFI co-related with the intensity of the protein detected by WB in the same sample, being lower for SK-Mel-147 which displayed fainter bands for CD63 and CD9.

### EV proteins can be detected in small amounts of minimally processed urine

**Figure 6:**
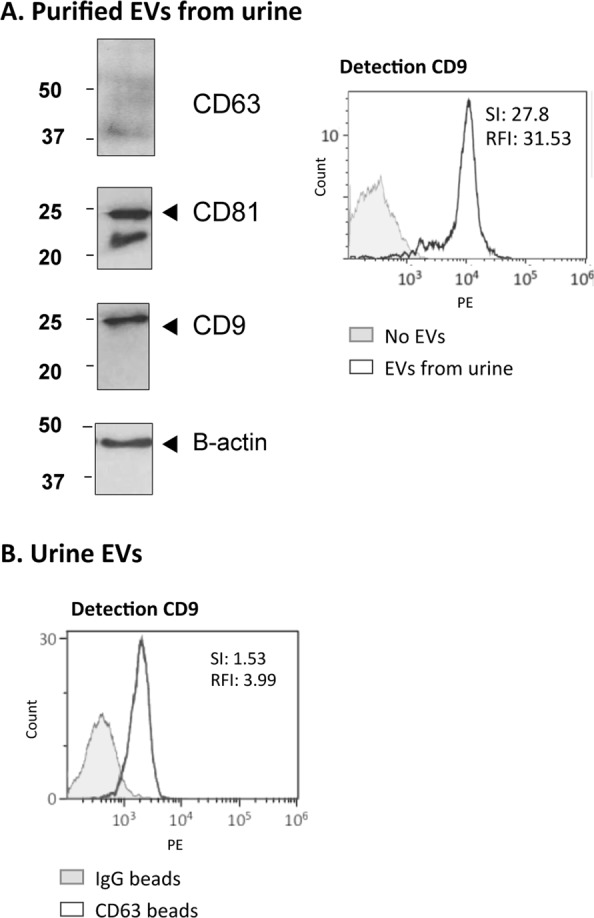
Detection of EVs from healthy donors urine. (**A**) Purified EVs from urine. 68 µg (6.8∙10^10^ particles) of EVs from healthy donors (Hansa Biomed) were analysed by Western Blot for detection of CD63 (1 h exposure), CD81 (10 min exposure), CD9 (10 s exposure) and β-actin (10 min exposure) (left). 10 µg (10^9^ particles) were captured onto anti-CD63-coated beads followed by detection with biotinylated anti-CD9 antibody by flow cytometry (right). (**B**) EVs contained in 500 µl of urine analysed by flow cytometry. 500 µl of healthy donor urine was pre-treated by mild reduction (see methods) and incubated with either anti-CD63- or IgG1-coated beads followed by detection with biotinylated anti-CD9 antibody by flow cytometry. Stain Index (SI) and the Relative fluoresce Index (RFI) are indicated inside each panel. A representative experiment out of 3 is shown.

Since the experiments above revealed a very high sensitivity for detection of EVs after pre-enriching by ultracentrifugation, we hypothesised that it would be possible to implement the assay in body fluids without previous enrichment of EVs. The basic assay of immune-capture on CD63 beads and detection using biotinylated-anti-CD9 was used first to detect EVs enriched by ultracentrifugation from healthy donor urine, with very high SI and RFI, 27.8 and 31.53 respectively (Fig. 6A). Next, only 500 µl of urine from healthy donors was analysed by flow cytometry. Although for most experiments, urine was pre-treated with mild reduction to eliminate Tamm-Horsfall protein (THP) aggregates, we could visualise EVs in urine samples in which only cell debris were eliminated by centrifugation at 400 × g (Supplementary Fig. 5A). EVs from 500 µl of urine were captured with anti-CD63 antibody and detected by flow cytometry using biotinylated-CD9 antibody. As negative control, IgG1-coated beads were incubated with 500 µl of urine from the same sample (Fig. 6B). The combination of anti-CD9 for both capture and detection also allowed visualization of the EVs by flow cytometry (Supplementary Fig. 5B).

**Figure 7:**
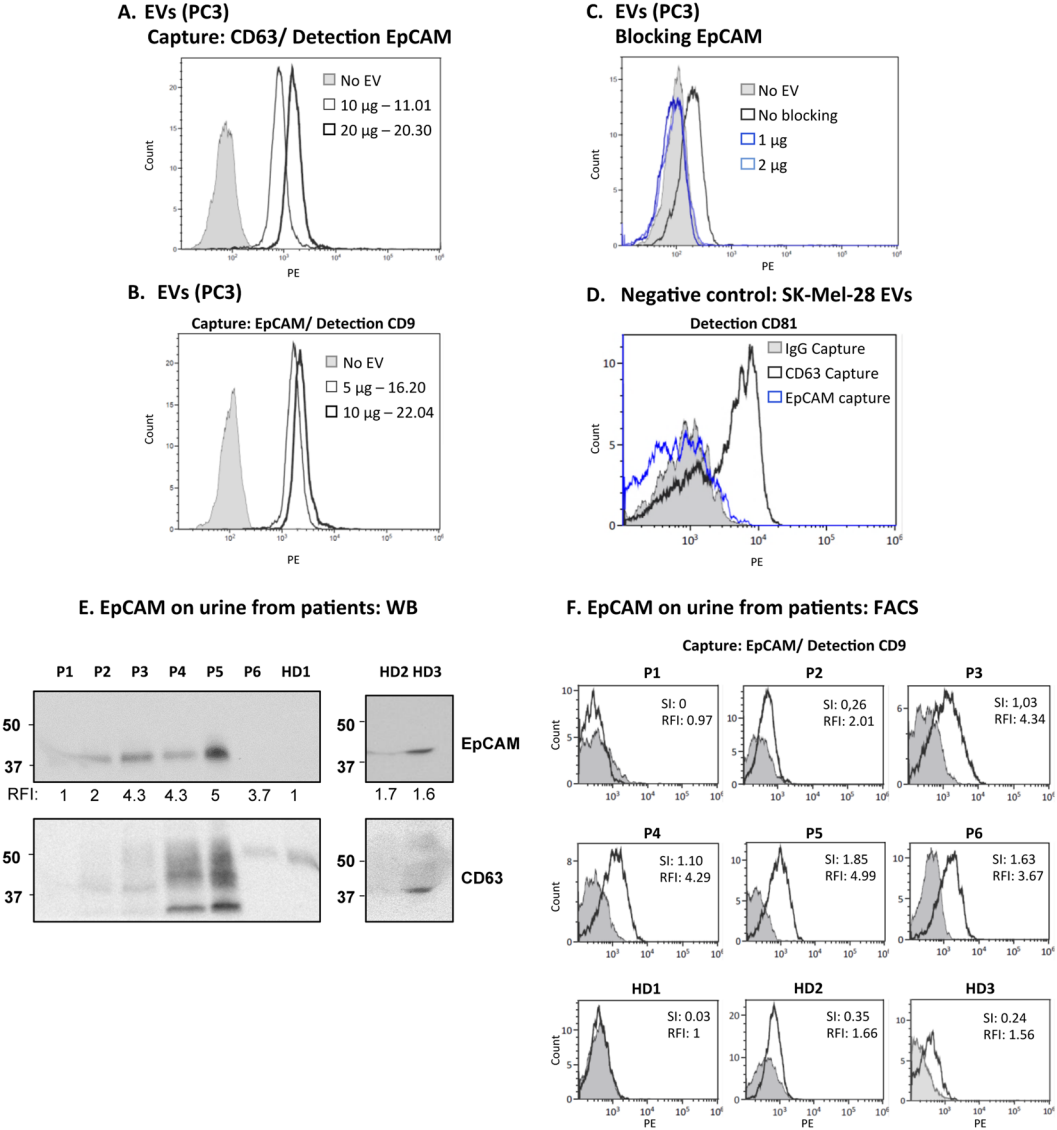
Direct detection of EpCAM in urinary EVs. (**A**) EpCAM detection on PC3-derived EVs. 10–20 µg (4.59–9.18∙10^9^ particles) of PC3-derived EVs were captured onto anti-CD63-coated beads followed by detection with biotinylated anti-EpCAM antibody by flow cytometry. (**B**) 5–10 µg (2.29–4.59∙10^9^ particles) of PC3-derived EVs were captured onto anti-EpCAM-coated beads followed by detection with biotinylated anti-CD9 antibody by flow cytometry. (**C**) Specificity of anti-EpCAM-coated beads: antibody blocking. To confirm the specificity of anti-EpCAM-coated beads, PC3-derived EVs were pre-incubated with anti-EpCAM antibody, previously to their incubation with microbeads. (**D**) Specificity of anti-EpCAM-coated beads: negative control. 1.78∙10^9^ particles of SK-Mel-28-derived EVs (EpCAM-negative cell line) were captured onto either anti-CD63-coated beads, anti-EpCAM- or IgG1-coated beads followed by detection with biotinylated anti-CD81 antibody by flow cytometry. (**E**) Detection of EpCAM by WB in EVs from 8 ml of urine. 8 ml of pre-treated urine from 3 healthy donors (HD) and 6 patients (P1- P6) were used to purify EVs by ultra-centrifugation and they were analysed by WB. The number under the EpCAM panel corresponds to the Relative fluoresce Index (RFI) in the flow cytometry experiment. (**F**) Detection of EpCAM by flow cytometry in EVs from 500 µl of urine. 500 µl of pre-treated urine were incubated with either IgG1 or anti- EpCAM-coated beads followed by detection with biotinylated anti-CD9 antibody and flow cytometry analysis. Three flow cytometry experiments were performed using the same patient samples and the results from a representative experiment are shown.

The ability to detect EVs in a small volume of urine together with the results showing that the capture antibody does not need to recognise an abundant protein for efficient immobilization of EVs, implies that all kind of different markers could be studied using this methodology. One of the most promising areas in the field of EVs consists in their use as tumour biomarkers. To formally prove this hypothesis, we decided to use EpCAM, a common marker found in epithelial carcinomas, in combination with tetraspanins. EpCAM is expressed in normal epithelial cells but is generally upregulated in carcinoma cells compared with healthy epithelia^34–36^ and the recruitment of this protein to EVs has been described in cell lines and patient biological fluids^37,38^. PC3 cells are positive for EpCAM^36^ and the protein was also detectable in cells and PC3-derived EVs by WB (Supplementary Fig. 1). Thus, several antibody combinations were tried next for detection by flow cytometry of EpCAM in EVs from prostate cancer cells. Firstly, PC3-derived EVs captured on anti-CD63 coated beads were analysed by flow cytometry using biotinylated anti-EpCAM antibody for detection and, then, using magnetic beads coated with anti-EpCAM antibody, followed by detection with anti-CD9 antibody (Fig. 7A,B). Both antibody combinations allowed detection of the EpCAM positive EVs with RFI values above 10. The specificity of EpCAM-coated beads was tested in blocking experiments using PC3-derived EVs (Fig. 7C). Also, specific binding was demonstrated by comparing the signals obtained with EVs from the EpCAM-negative cells, SK-Mel-28 (Supplementary Fig. 4), on beads coated either with EpCAM, CD63 or IgG1 (Fig. 7D).

The expression of EpCAM was analysed directly in urinary EVs from 9 donors that attended the urology clinic with prostate swelling or suspected cancer^36,39^. Urine samples were used for parallel determinations of EpCAM on EVs: (1) EVs enriched by ultracentrifugation from 8 ml of starting urine were analysed by Western Blot (Fig. 7E); (2) 500 μl of urine were analysed by capture on anti-EpCAM coated beads followed by detection using biotinylated anti-CD9 by flow cytometry (Fig. 7F). Expression of EpCAM was detected by flow cytometry in 7 of the 9 samples available, while only in 6 samples this marker was detectable by WB. Thus, in these experiments, flow cytometry allowed detection of EpCAM on EVs with higher sensitivity than WB, confirming that this method can be used to phenotype EVs even in small amounts of biological samples and thus allow larger screening studies. Our results provide a proof-of-concept for direct detection of tumour markers in EVs from urine samples by immune capture followed by flow cytometry.

## Discussion

The field of EVs has benefited in the last years from intense research which has very rapidly yielded information on their molecular content in health and disease. As a result, much interest has been attracted to the possible use of EV-expressed molecules as biomarkers in a number of pathologies. Although EVs can be very accessible in biological fluids, their low concentration in particular EV populations and/or specific EV molecules together with the heterogeneity in their origin, and thus in molecular composition, make it difficult to screen a high number of samples for biomarker analysis and validation. Thus, it is important to develop methodologies that would allow such high throughput studies using, when possible, generally available techniques. Here, we describe a highly sensitive reproducible method for immune capture of EVs followed by conventional flow cytometry detection that allows characterization of small amounts of vesicles, even directly from as little as 500 μl of minimally processed urine. Our results demonstrate that using antibody-coated magnetic microbeads it is possible to detect non-abundant proteins on EVs in a wide concentration range, with a limit of detection below that of Western Blotting. Further, the use of conventional flow cytometry provides the advantage of allowing easy implementation in nearly any research institute or hospital laboratory.

Several immune-capture methods are currently under study for the characterization of EV composition. In fact, several companies commercialize kits to perform such types of assays. The use of antibodies to bind a specific marker allows, in principle, to distinguish vesicles originated from different cells, compartments or organs and to selectively study different vesicle subpopulations, as well as to avoid the analysis of contaminants accumulated during ultracentrifugation. However, the experimental strategies using immunocapture vary markedly, and the sensitivity of different types of assays can be critically affected at different steps. For this reason, we considered here the theoretical critical parameters necessary for a reliable detection by flow cytometry and carefully compared the conditions to perform the test. Although changes in the conditions of temperature and incubation time could clearly affect binding of EVs to the microbeads, one of the main factors necessary to improve the assay sensitivity relies on the relative size and number of microbeads and vesicles in the analysis.

The assay reported here allows detection of EV populations (as Relative Stain Index, SI, and Relative Fluorescence Index, RFI) both when using tetraspanins, as general EV markers, or by using a specific marker of epithelial cells, EpCAM. Moreover, for effective capture of EVs, it was not necessary to use the more abundant antigens detected by WB. Because multiple proteins can be exposed on the vesicle, binding is favored by avidity and, thus, if the antibody’s affinity and specificity are good, the abundance of the antigen is not a limiting factor for capture. This is demonstrated by the effective capacity of the anti-CD63 antibody used here to capture EVs, even if this protein was less abundant than other tetraspanins in the sample.

The method for EVs immunocapture and flow cytometry analysis described here has higher sensitivity than WB for individual proteins and allows the detection of several combinations of epitopes. While 6.8·10^9^ particles were needed for WB detection of CD63, 1.37·10^7^ particles were detected by flow cytometry, increasing the possibility of visualising almost any marker. Moreover, the method provides the advantage of making possible the study of different epitopes on the captured vesicles allowing confirmation of the presence of multiple markers in a population of EVs. This could be further developed for the study of more protein markers, using combinations of several fluorescent antibodies and provides the possibility of easily adapting to this method a multiplex analysis, including microbeads coated with different antibodies, taking advantage of the fluorescence feature of the beads.

The high sensitivity of the assay allowed detection of protein markers directly in urine. This direct detection opens the way to compare data among different laboratories with high reproducibility, since the heterogeneity introduced by EV isolation techniques could be eliminated. We demonstrated the possibility of detecting general markers of EVs in urine from healthy donors and provided the proof-of-concept for the detection of cancer markers directly in urine of prostate cancer patients. The methodology reported here can be easily adapted to examine larger cohorts of patients and different tumour markers to establish the link between EV-expressed proteins and disease progression. This method complements the one recently reported by Sharma *et al*.^28^, in which EVs isolated from melanoma patients plasma were detected by flow cytometry using a specific marker for melanoma. Further, the method reported here shows increased sensitivity with RFI values above 30 for purified EVs and allowed detection without previous enrichment. Therefore, the use of flow cytometry for routine screening of biological samples can become a reality for patient classification and treatment follow-up. Further normalization studies should be needed for the screening of patient urine, including: 1/Normalization of dilute versus concentrated urine due to donor hydration. This can be addressed measuring creatinine which is constantly released under normal kidney filtration function; 2/Normalization of donor-to-donor variation, can be addressed using the IgG beads which can detect variations in autofluorescence or any contaminant causing non-specific binding; 3/changes in the amount of EVs released by each donor due to their particular patho-physiological events. This requires clinical research on markers that are more or less abundant, or completely absent, in the context of different diseases.

In conclusion, we have maximized fluorescence detection on immunocaptured EVs to develop a robust and reproducible assay allowing protein phenotyping with high sensitivity. We also provide the proof-of-concept for the detection of EV markers, such as tetraspanins and EpCAM directly in urine.

## Materials and Methods

### Cells lines and reagents

Cell lines from prostate (PC3), renal carcinoma (HEK293) and melanoma (Ma-Mel-55^40^, SKMel147, SKMel28) were grown in RPMI 1640 with 10% fetal bovine serum (FBS), L-Glutamine 1 mM, Penicillin and Streptomycin 100 μg/ml, Sodium Pyruvate 1 mM, non-essential aminoacids 0.1 mM and HEPES 10 mM, at 37 °C. Bladder cancer cells (RT-112) were cultured in DMEM with the same supplements. The human breast carcinoma cell line SUM159 was cultured in DMEM/F-12 (Life Technologies, Invitrogen, Carlsbad, CA), supplemented with 5% FBS, nonessential amino acids, 5 μg/ml insulin and 1 μg/ml hydrocortisone. The breast cancer cell line MCF-7 was cultured in DMEM, 10% FBS, 1% penicillin/streptomycin, Sodium Pyruvate 1 mM and 10 μg/ml insulin.

Antibody-coated magnetic beads were obtained from the Exostep™ kit (Immunostep, Ref: ExoS-25-U) or prepared by incubating magnetic fluorescent beads either from Luminex (MagPlex Microsphere, Ref: MC10012) or Bangs (QuantumPlex M SP Carboxil, Ref: 251A) with purified anti-CD63 (Clone TEA3/18), purified anti-CD9 (Clone VJ1/20) or anti-EpCAM (Clone VU-1D9) antibodies from Immunostep, S.L. Magnetic fluorescent beads were coated with high density carboxyl functional groups on the surface, these groups were used to covalently conjugate antibodies through their primary amines by two-step EDC/NHS protocol producing a stable amide bond.

Unless otherwise stated, all chemicals were purchased from Sigma-Aldrich.

### EV extraction and isolation

For comparison of different cell lines, EVs were isolated from cell culture supernatants by sequential centrifugation as previously described^41^. Cells were cultured for 72 hours to 7 days in a medium containing 1% exosome-depleted FBS (prepared by centrifugation for 20 h at 100,000 × g). After the final centrifugation at 100,000 × g, EVs were resuspended in Hepes-buffered saline buffer (HBS: 10 mM HEPES pH 7.2, 150 mM NaCl). For some experiments, lyophilized exosome standards from PC3 cell culture supernatant were purchased from HansaBiomed or Immunostep, S.L. (Ref: ExoPC3). Lyophilized exosomes from human urine of healthy donors were purchased from HansaBiomed. Lyophilised EVs from PC3 cell culture supernatant were resuspended at 1 µg/µl.The protein amount, corresponding to the μg of EVs, has been calculated by BCA assay, which assesses the total protein content.

### EV quantitation by nanoparticle tracking analysis

Concentration and size of EVs were determined by nanoparticle tracking analysis (NTA) in a Nanosight NS500 (Malvern Instruments Ltd, Malvern, UK) equipped with a 405 nm laser. The settings used were: Camera level:12, Focus between −15 and +15, Threshold: 10, Capture: 60 s., Number of Captures: 3, Temperature 25 °C. These experiments were carried out in the laboratory of Dr. H Peinado, at the Spanish National Centre for Oncological Research (CNIO).

### Western Blot

Equal amounts of EVs (6.8 · 10^9^ particles) were loaded in 10% SDS-PAGE gels, either under reducing or non-reducing conditions, as indicated in the experiments, and transferred to membranes with Trans-Blot® Turbo™ Transfer Packs (Biorad). Membranes were blocked using 5% non-fat dry milk in PBS containing 0.1% Tween 20 (PBS-T). Primary antibody was incubated for 1 h in PBS-T and, after washing, membranes were incubated with the appropriated secondary antibody. Proteins were visualized using the ECL system (Amersham Biosciences). Antibodies used were: monoclonal anti β-actin produced in mouse (Sigma) at 0.13 µg/ml; purified mouse monoclonal anti-CD9 (MEM62), -CD63 (MEM259) and -CD81 (MEM-38) antibodies (kind gifts from Vaclav Horejsi, Croatia); horseradish peroxidase-conjugated goat anti-mouse antibody (Dako). Rabbit polyclonal anti-calreticulin (Novus Biologicals) was used for WB at 4 µg/ml and biotinylated anti-EpCAM (clone VU-1D9) at 1 µg/ml.

### EV flow cytometry

EVs were incubated with 6000 antibody-coated beads in 100 μl of PBS containing 1% casein for 18 h, in a 5 ml tube without agitation at room temperature (RT). After the binding step, beads were stained with either anti-CD63 (Clone TEA3/18), anti-CD81 (MEM-38) or anti-CD9 (Clone VJ1/20) antibodies (Immunostep, S.L.), either biotinylated or PE-conjugated. After antibody binding, beads were washed with filtered PBS containing 1% BSA, and recovered using a Magnetic Rack (MagneSphere(R) Mag. Sep. Stand 12-hole, 12 × 75 mm (Promega, Ref Z5343). When using biotinylated antibody, a step incubating with streptavidin-PE (Immunostep, S.L.) was added followed by bead washing with PBS-1% BSA. When analysing urine samples, 500 μl of urine supernatant (see below) were directly incubated with 3000 microbeads. Samples were analyzed using either Gallios or Cytomics FC 500 (Beckman Coulter) or FACSCalibur Flow Cytometers (Becton Dickinson) and data were analysed using Kaluza (Beckman Coulter) and FlowJo (Tree Star, Inc). Single beads were gated in Forward Scatter in the region corresponding to 6 µm (established using calibration beads (FlowCheck Pro^TM^ fluorospheres, Beckman Coulter), excluding bead doublets and non-bead events in FSC/SSC and selecting beads using the corresponding channel of fluorescence. The percentage of single beads was above 95%.

### Urine samples

Urine from 3 healthy donors and 8 patients with enlarged prostate was collected at the Urology Service at University Hospital La Paz (Madrid). Of these donors, two were excluded from the analysis: one did not contain EVs by WB; the second had large amounts of macroscopic aggregates in the urine after a first centrifugation at 200 × g. All volunteers signed an informed consent form with the ethical approval of the Institutional Review Board and local ethical committees (CEI La Paz Hospital HULP-PI 2978). The study conformed to the principles expressed in the Declaration of Helsinki.

10 ml of urine were centrifuged at 400 × g to remove cells and Tamm-Horsfall protein (THP) aggregates, this initial pellet was treated for 10 min with 28 µl of 100 mM DTT, and after centrifugation at 400 x g the supernatant was transferred to the previous 10 ml of urine supernatant (final concentration of DTT 0.28 mM).

## Supplementary information

Supplementary Figures
