# Supplementary material for: High sensitivity detection of extracellular vesicles immune-captured from urine by conventional flow cytometry
Source: Sci Rep. 2019 Feb 14;9:2042. doi: 10.1038/s41598-019-38516-8 (PMC6376115; doi:10.1038/s41598-019-38516-8)
Supplement: Supplementary file 1 — Supplementary Figures [file 41598_2019_38516_MOESM1_ESM.pdf]

High sensitivity detection of extracellular vesicles immune-captured from urine by conventional flow cytometry

Carmen Campos-Silva, Henar Suárez, Ricardo Jara-Acevedo, Estefanía Linares-Espinós, Luis Martinez-Piñeiro, María Yáñez-Mó and Mar Valés-Gómez

SUPPLEMENTARY INFORMATION

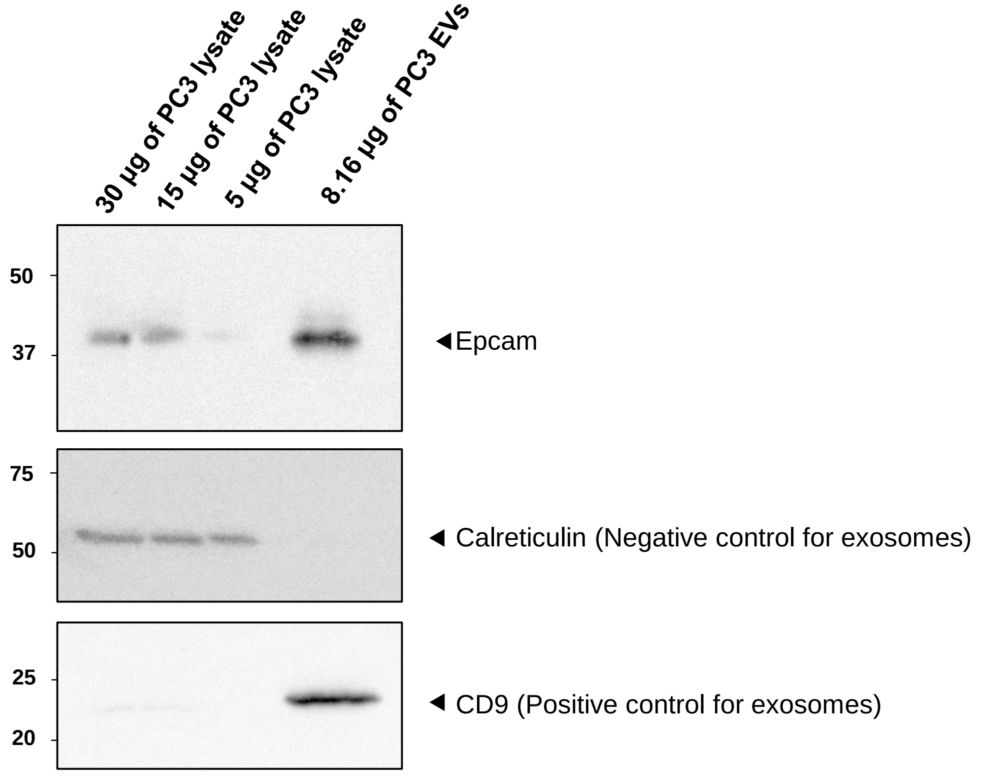

Supplementary Figure 1. **Characterization of PC3-derived EVs; comparison with cell lysates.** Different amounts of cell lysates (30 µg, 15 µg and 5 µg), prepared in 1% NP-40 lysis buffer, spun to eliminate nuclei, and $6.8{\cdot}10^9$ particles (8.16 µg) of EVs obtained by ultracentrifugation from PC3 cell culture supernatant were loaded on SDS-PAGE and immunoblotted for EpCAM (5 s), calreticulin (1 s) and CD9 (5 s)]. One gel was loaded under non-reducing conditions, for the detection of the 3 proteins.

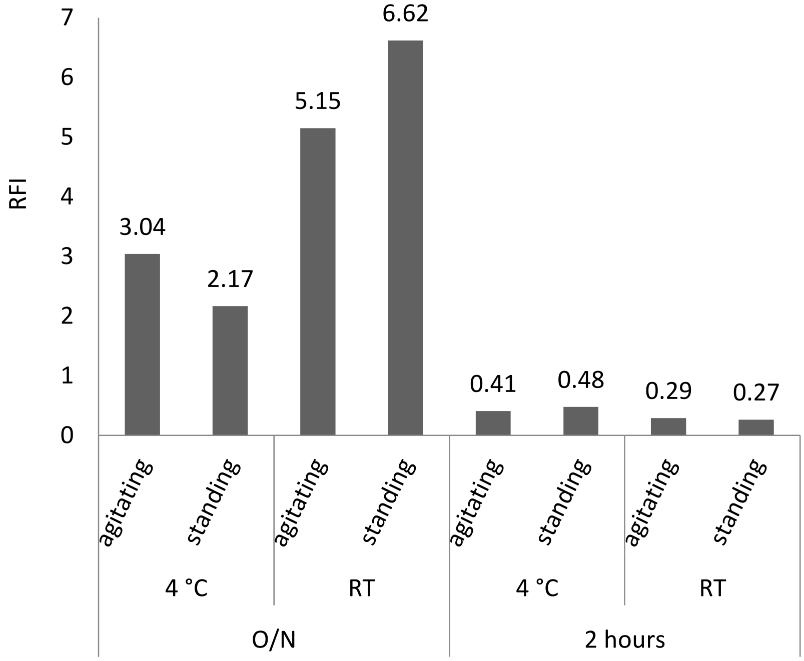

Supplementary Figure 2. **Comparison of different temperature, time, and agitation conditions for the immune-capture step.** 1.8 µg of PC3-derived EVs (8.26 x 10^8 particles) were captured onto anti-CD9 coated beads followed by detection with PE-conjugated antibody directed against CD81 at the conditions indicated in the x-axis. After flow cytometry, the Relative Fluorescence Index (RFI) was calculated and plotted. The RFI value obtained for each condition is indicated on top of each column. This is a representative experiment out of 3.

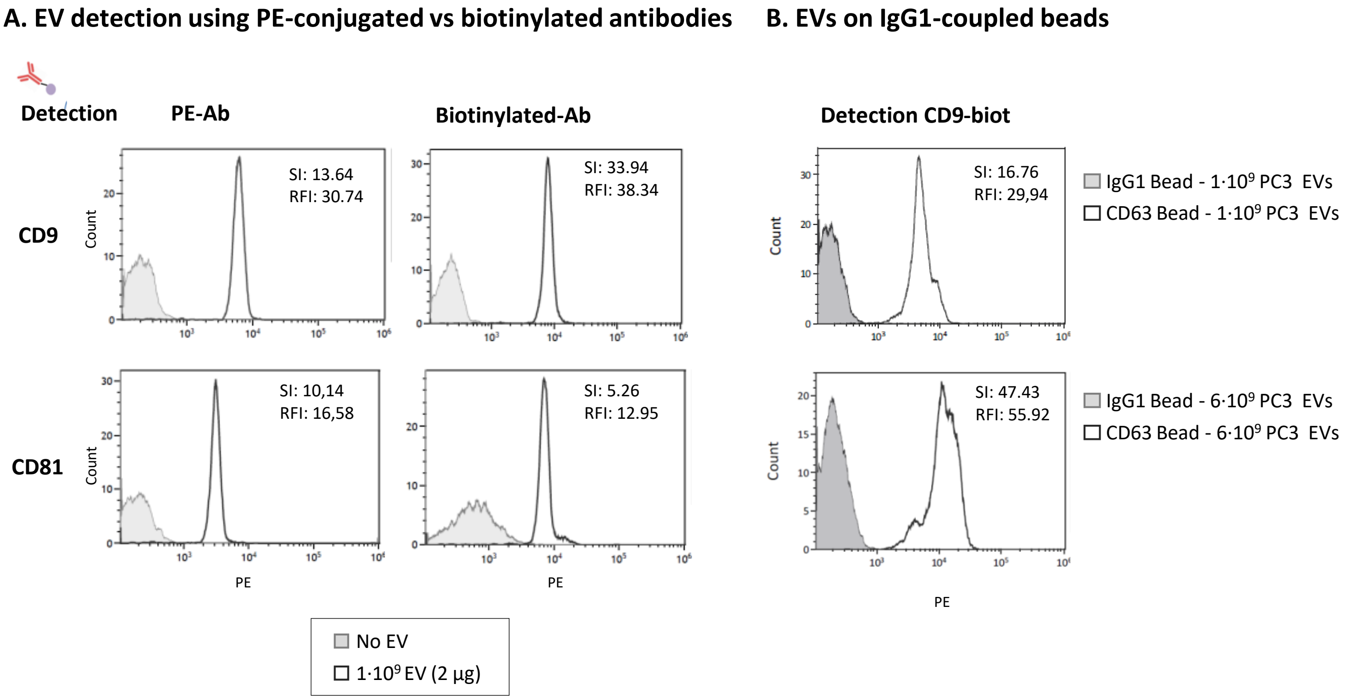

Supplementary Figure 3. **EV detection by flow cytometry. A. EV detection using PE-conjugated vs biotinylated antibodies.** 2 µg (10^9 particles) of PC3-derived EVs were captured onto anti-CD63 coated beads followed by detection with either PE-conjugated or biotinylated antibody directed against CD81 or CD9, as indicated. The samples were analysed by flow cytometry. **B. IgG1-coupled beads do not bind EVs.** Increasing amounts [$1 \cdot 10^9$ particles=1.3 µg PC3 EVs (upper plot); $6 \cdot 10^9$ particles=7.8 µg PC3 EVs (bottom plot)] of PC3-derived EVs were captured onto either IgG1- or anti-CD63-coated beads followed by detection with biotinylated antibody directed against CD9 and analysed by flow cytometry.

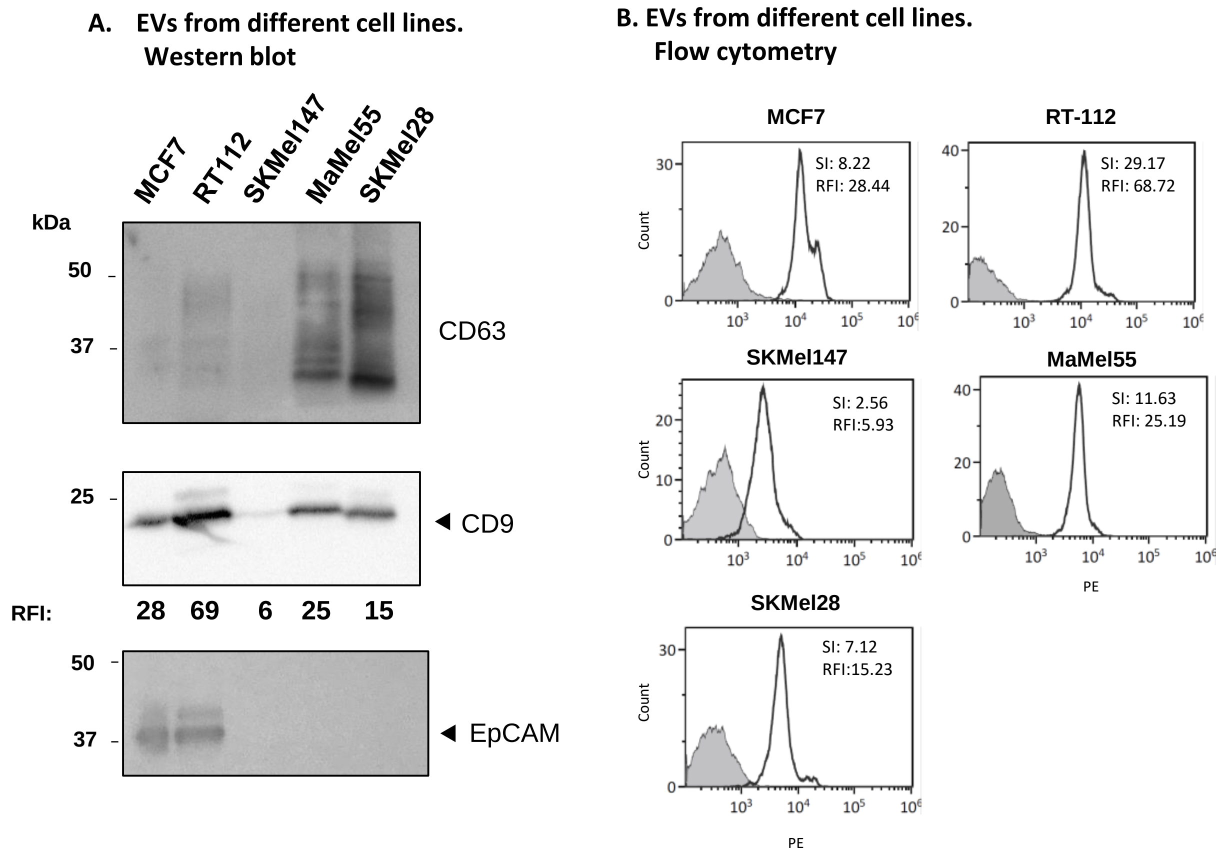

Supplementary Figure 4. Validation of the assay in EVs from different sources. A. Western blot of EVs from different cell lines. EVs were isolated from 30 ml of cell culture supernatant (10^7cells) from the indicated tumour cell lines and resuspended in HBS in a final volume of 90 µl. $6.8 \cdot 10^9$ EVs were analysed by Western Blot to compare relative amounts of different tetraspanins as indicated. The number under the CD9 panel corresponds to the Relative fluoresce Index (RFI) in the flow cytometry experiment. **B. Flow cytometry of EVs from different cell lines.** $3 \cdot 10^9$ EVs were captured onto anti-CD63-coated beads followed by detection with biotinylated anti-CD9 antibody in flow cytometry. Stain Index (SI) and the Relative fluoresce Index (RFI) are indicated in each panel.

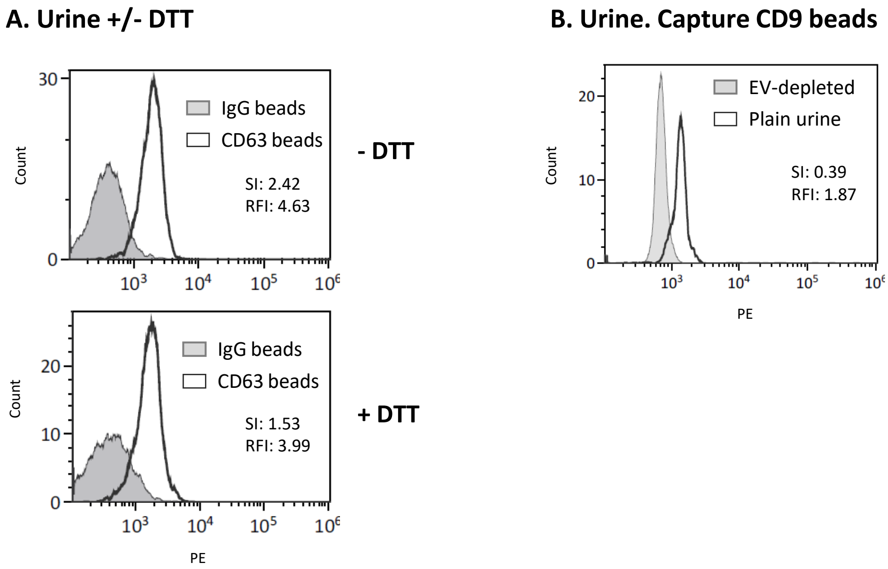

Supplementary Figure 5. **Direct detection of EV markers in urine**. **A. Effect of mild reduction on detection of tetraspanins by flow cytometry.** Urine samples from healthy donors were either pre-treated with a reduction step to eliminate Tamm-Horsfall protein (THP) protein aggregates (+DTT) or used after a 400 x g centrifugation for immune capture on antibody-coated beads (-DTT). 500 µl of urine were incubated with either anti-CD63- or IgG1-coated beads followed by detection with biotinylated anti-CD9 antibody in flow cytometry. **B. Capture of urine EVs on anti-CD9-coated beads.** 500 µl of pre-treated urine were incubated with anti-CD9-coated beads followed by detection with biotinylated anti-CD9 antibody in flow cytometry. As a negative control, urine depleted of EVs by 100000 x g ultracentrifugation was used. Stain Index (SI) and the Relative fluoresce Index (RFI) are indicated in each panel.
